# Tackling wicked problems in infection prevention and control: a guideline for co-creation with stakeholders

**DOI:** 10.1186/s13756-016-0119-2

**Published:** 2016-05-21

**Authors:** Anne F. G. van Woezik, Louise M. A. Braakman-Jansen, Olga Kulyk, Liseth Siemons, Julia E. W. C. van Gemert-Pijnen

**Affiliations:** Center for eHealth and Wellbeing Research; Department of Psychology, Health and Technology. Faculty of Behavioral, Management and Social Sciences, University of Twente, Enschede, Netherlands; Department of Medical Microbiology, University of Groningen, University Medical Center Groningen, Groningen, Netherlands

**Keywords:** eHealth, Guideline, One health, Stakeholder, Wicked problem, Zoonosis

## Abstract

**Background:**

Infection prevention and control can be seen as a wicked public health problem as there is no consensus regarding problem definition and solution, multiple stakeholders with different needs and values are involved, and there is no clear end-point of the problem-solving process. Co-creation with stakeholders has been proposed as a suitable strategy to tackle wicked problems, yet little information and no clear step-by-step guide exist on how to do this. The objectives of this study were to develop a guideline to assist developers in tackling wicked problems using co-creation with stakeholders, and to apply this guideline to practice with an example case in the field of infection prevention and control.

**Methods:**

A mixed-method approach consisting of the integration of both quantitative and qualitative research was used. Relevant stakeholders from the veterinary, human health, and public health sectors were identified using a literature scan, expert recommendations, and snowball sampling. The stakeholder salience approach was used to select key stakeholders based on 3 attributes: power, legitimacy, and urgency. Key values of stakeholders (*N* = 20) were derived by qualitative semi-structured interviews and quantitatively weighted and prioritized using an online survey.

**Results:**

Our method showed that stakeholder identification and analysis are prerequisites for understanding the complex stakeholder network that characterizes wicked problems. A total of 73 stakeholders were identified of which 36 were selected as potential key stakeholders, and only one was seen as a definite stakeholder. In addition, deriving key stakeholder values is a necessity to gain insights into different problem definitions, solutions and needs stakeholders have regarding the wicked problem. Based on the methods used, we developed a step-by-step guideline for co-creation with stakeholders when tackling wicked problems.

**Conclusions:**

The mixed-methods guideline presented here provides a systematic, transparent method to identify, analyze, and co-create with stakeholders, and to recognize and prioritize their values, problem definitions, and solutions in the context of wicked problems. This guideline consists of a general framework and although it was applied in an eHealth context, may be relevant outside of eHealth as well.

## Background

In today’s society of human dominance, international travel, urban crowding, and other human behaviors associated with distorting ecological balance in the world, infection prevention and control can be seen as a societal challenge, a multifaceted problem, and an outright ‘wicked problem’ [1]. Wicked problems were first described by Rittel and Webber as a category of public policy problems that, in contrast to ‘tame problems’, are difficult to be clearly defined, are influenced by complex social and political factors, and are never solved [2]. The authors outline ten distinguishing characteristics of wicked problems, of which three are particularly applicable to infection prevention and control [2–4]:

*1 No consensus regarding the problem definition.* No exhaustive problem formulation can be given, as an understanding of the problem is based on the understanding of the solutions to the problem. Because there are often many different solutions to a wicked problem, there are also many different problem definitions that can be formulated.

*2 Involvement of multiple, often independent stakeholders*. Wicked problems are characterized by many different stakeholders with a stake in the problem, often with different or even conflicting views on what the problems and solutions are. As a result, there is a high risk for conflict. It is impossible to please everyone and trade-offs are necessary to somehow address the needs of those involved.

*3 No clear cut “stopping rule”.* As there is no clear problem definition and no clear solution, there are also no criteria to evaluate whether a solution has been found. This in turn makes it unclear when efforts to tackle wicked problems can be ceased. What is a job well done to one group of stakeholders can be an unacceptable progression according to others. It is likely that efforts will be terminated because of external issues such as a lack of time or money.

Wicked problems, in our view, are continuous, dynamic problems that require multi-dimensional, flexible solutions. Wicked problem solutions involve compromises between competing values, call for a multidisciplinary approach, and depend on collaboration between those parties involved [5]. Considering the fact that stakeholders have different views on the problem and its solution [2, 4], stakeholders will have different important insights to contribute.

According to stakeholder theory, an organization is at the center of a network of stakeholders, with stakeholder being defined as ‘any group or individual who can affect or is affected by the achievement of the organization’s objectives’ (p. 46) [6]. Translated to the context of wicked problems, this implies that scientists working towards tackling these kind of problems have to take the relevant stakeholders in the existing stakeholder network into account, as unsupportive, uninvolved, and even hostile stakeholders can hinder sustainable implementation of the research project [7, 8]. In the context of eHealth development, the importance of involving stakeholders in research projects is becoming more and more evident [7–12]. In our research, several eHealth interventions have been conducted in the field of infection prevention and antibiotic stewardship, with findings suggesting promising effects of stakeholder involvement in the development of these projects, but further application of their methods and validation of their findings is needed [11, 12]. In a co-creation approach, researchers view stakeholders as active contributors of value and collaborate with them not only in the implementation phase but also in the design phase [11], thereby generating positive effects such as increased stakeholder commitment to and ownership of the project [10, 13]. Although the benefits of co-creating with stakeholders are clear, working with stakeholders comes with several challenges that need to be overcome, such as working with different values and priorities and investing a considerable amount of time in building relationships [14].

Although the need to involve stakeholders when tackling wicked problems is clear, the required approach and methodology to do so are not. Even though many different techniques exist to identify stakeholders and to assign importance to them [8, 15], there is little information and no clear step-by-step guide available on how to co-create with stakeholders successfully. Especially in the field of wicked problems, very few studies have been conducted with stakeholders. Signal and colleagues did use a stakeholder-driven approach, but do not elaborate in detail on how they did this and how others could do the same [16]. Van Limburg et al. [12] were to our knowledge the first who proposed a guideline on how to involve and co-create with stakeholders. However, a limitation to this framework is that it is only demonstrated in one example case. Further validation of its generic use as a complete framework for other projects, particularly in the context of wicked problems, is warranted.

### Example case: zoonoses prevention and control

The majority (60.3 %) of all emerging and re-emerging infectious diseases are zoonotic in nature [17]. A zoonotic infection is an infection that can be transmitted by animals to humans [18]. Zoonoses control and prevention are wicked problems as they fit the three characteristics of wicked problems (no consensus regarding a problem definition, involvement of multiple stakeholders, and no stopping rule) [2, 4]. In addition, they (A) have economical, sociological and political implications [19, 20], (B) consist of a complex stakeholder network comprising of many stakeholders from different sectors across different hierarchical levels (from local veterinarians to governmental decision makers) with strongly held different or even opposing views, and (C) harbor an implementation context which is a dynamic field in which new initiatives start rapidly alongside each other which generates competition. The World Health Organization recognized that interdisciplinary collaboration between different sectors involved (including veterinary, human and public health) is essential [19], which is in line with the global “One Health approach” that emphasizes interdisciplinary collaboration and communication in order to prevent and control zoonoses [21]. However, in reality a lack of collaboration between these three sectors, uncertainties about their respective tasks and responsibilities for risk communication, and poor knowledge of the general public about zoonoses negatively affect current risk communication strategies in the Netherlands [22].

In order to help tackle the wicked problem of zoonoses prevention and control in the Netherlands, we are involved in the development of an online platform in line with the One Health approach, called the “eZoon” platform. The aims of the platform are to support collaboration and risk communication on non-alimentary zoonoses between the veterinary, human health, and public health sectors in the Netherlands. eZoon will be an online information-, education- and communication platform aimed at the general public and professionals working in these three sectors. Although the exact content and design of the platform are yet to be co-created with stakeholders and end-users, the platform will include a smart Question and Answer system aimed at informing the general public about zoonoses and a serious game to educate professionals on communication and collaboration. By involving stakeholders and attending to their needs, we tackle the three characteristics associated with wicked problems by 1) finding out what different stakeholders see as problems with and solutions to the prevention and control of zoonoses, 2) attending to stakeholder needs in order to increase cooperativeness, and 3) finding out what stakeholders see as solutions in order to identify when efforts can be ceased.

The aim of this paper is twofold. With it often being difficult to develop suitable strategies, solutions or innovations in the complex context of wicked problems like infection prevention and control and no guidance currently exist, our first objective is to develop an evidence-based, step-by-step guideline that can be used by anyone looking for a structured, evidence-based way to work in this context. This guideline is based on prior research on infection prevention and control and builds on the stakeholder-centered framework proposed by van Limburg et al. [12]. In addition, we will focus on these authors’ recommendations by using an integrative mixed-method approach to identify and prioritize stakeholders. Our second objective is to illustrate how to work with this guideline by demonstrating an example case of our own research in the field of One Health and zoonoses. This guideline covers the first step of how to collaborate with stakeholders, of which the results will guide further development of the project. By involving stakeholders and inquiring about their values, their perspective on the problem and possible solutions, we will address the three characteristics associated with wicked problems as described above.

## Methods

**Fig. 1:**
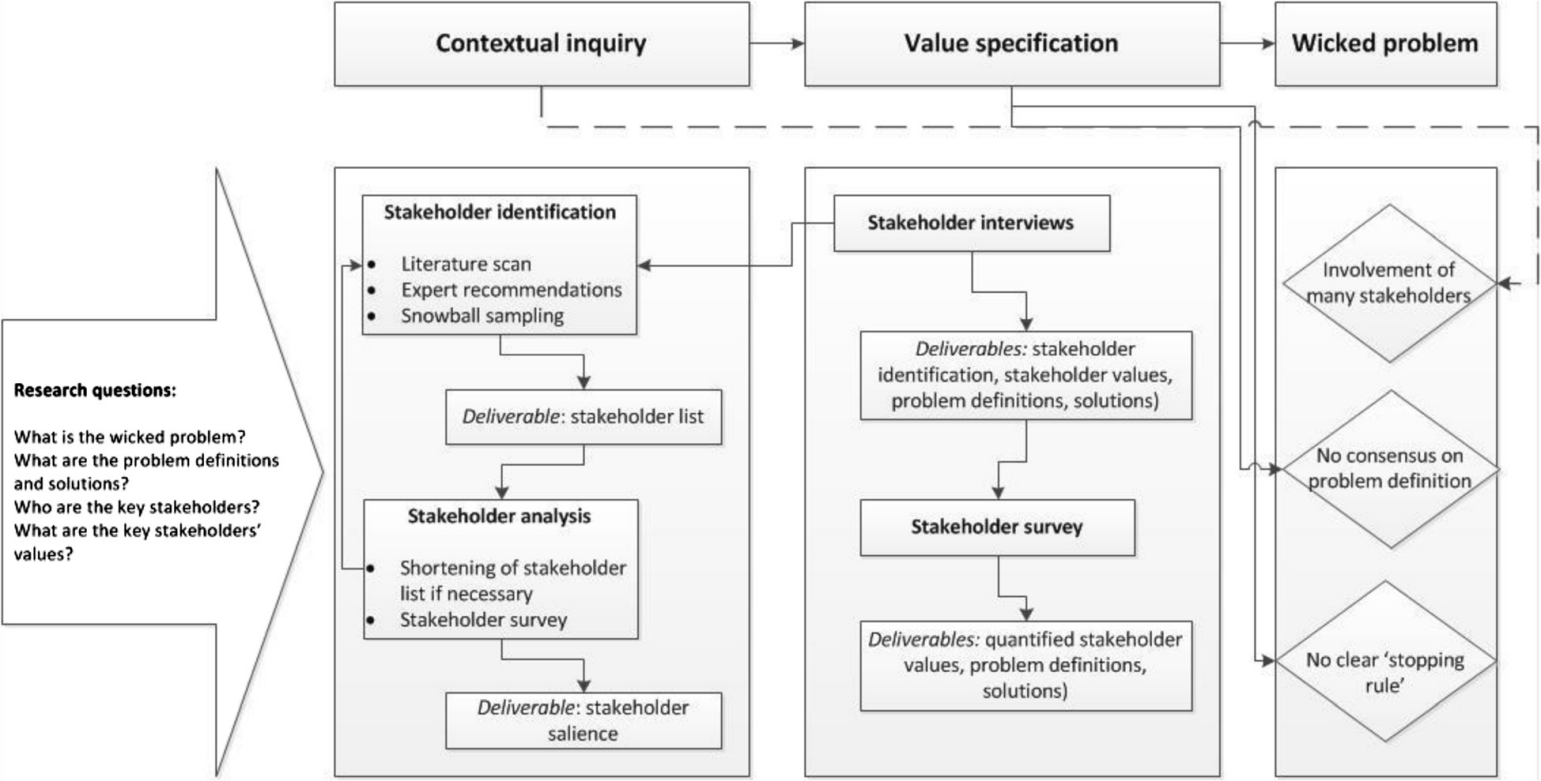
Process map describing the steps of the proposed guideline and their relation to wicked problems

The stakeholder-centered approach as outlined in this article builds upon the framework proposed by van Limburg et al. [12] who reflected on co-creating with stakeholders in eHealth development. The framework comprises of a mixed method approach consisting of the integration of both quantitative and qualitative research involving stakeholders and end-users. The first two phases of the Center for eHealth Research (CeHRes) roadmap will be applied to perform a contextual inquiry of the problem and to specify values of stakeholders and end-users, see [10, 12] and the CeHRes roadmap wiki [23] for a detailed description. For a visual representation of the proposed guideline, see the process map below (Fig. 1), and for a step-by-step guide, see Table 1. As can be seen in Fig. 1, the phases of contextual inquiry and value specification are not mutually exclusive and it is possible that the researcher is shifting between the two phases rather than moving on from contextual inquiry to value specification in a strict, static manner. New insights in the value specification phase may spark new stakeholders to be identified.

**Table 1:** Step-by-step guideline for stakeholder involvement and co-creation when tackling wicked problems

Step	Action
	Contextual inquiry:
1	Conduct a literature scan on the wicked problem of interest to get acquainted with its problem definitions, solutions, and (key) stakeholders. Both scientific and non-scientific literature, such as governmental reports, should be used.
2	Create an initial stakeholder list based on the literature review.
3	Involve at least two field experts to validate the list of stakeholders and to gain practical insights (now and further along in the project).
4	Involve and subsequently interview stakeholders from a range of different organizations with a variety of backgrounds in order to find missing stakeholders and to get an understanding of the different perspectives on the wicked problem.
5	If the complete list of stakeholders is too long, repeat steps 1, 3 and if possible 4 to shorten the list.
6	Let stakeholders rank the final stakeholder list to find out which stakeholders are perceived as key stakeholders.
	Value specification:
7	Conduct qualitative interviews or focus groups with key stakeholders with different backgrounds and hierarchical positions to get a better understanding of their values, needs, and perspectives on what the wicked problem is and how it can be solved. Take into account that each stakeholder has a unique expertise, so it is unlikely that a one-size-fits-all interview schedule will be suitable.
8	Transcribe and code transcripts from the interviews and/or focus groups to extract key values.
9	Validate stakeholders’ key values by sending a survey to all involved stakeholders.
10	Use all generated output to guide the subsequent phases of the process, such as the design phase.

### Contextual inquiry

Because contextual inquiry sheds a light on who is involved, how they are involved, and how important they are, the knowledge gained in this phase tackles the characteristic of involvement of multiple independent stakeholders associated with wicked problems.

*Stakeholder identification:* We chose a mixed methods approach consisting of a literature scan, expert recommendations and snowball sampling to identify all relevant stakeholders. In this case, a stakeholder was defined as ‘any group or organization that could affect or is affected by risk communication of zoonoses in the Netherlands’.

- Literature scan: In order to obtain an initial list of relevant stakeholders, an exploratory literature search concerning risk communication of zoonoses in the Netherlands was performed [22, 24–26]. This consisted of both scientific literature, see [24] for an example, as gray literature such as official governmental reports [22, 25, 26]. It helped the research team get familiarized with the field of zoonoses, the risk communication structure in the Netherlands, and stakeholders.
- Expert recommendations: Two experts, working in the infectious disease domain and the public health sector respectively, validated the stakeholder list and gave recommendations.
- Snowball sampling with stakeholders: Nine stakeholders were consulted to find missing stakeholders and to get initial information about who were considered to be key stakeholders. Stakeholders were interviewed from a wide range of sectors (including veterinary, human and public health organizations) and hierarchical positions (ranging from general practitioners and veterinarians working in a local practice to governmental policymakers) to ensure the interviewed stakeholder would have sufficient combined knowledge of the stakeholder field.

We used the same mixed method approach (literature scan, expert recommendations, snowball sampling) to narrow our total list of stakeholders down to a manageable list with the purpose of shortening the amount of time stakeholders would need to invest when rating the stakeholder list. We reread official reports (literature scan), held group discussions with experts (expert recommendations), and asked a stakeholder to give feedback on our stakeholder list (snowball sampling). In addition, we consulted an expert in survey efficiency to determine what was considered to be a manageable list. The final list of stakeholders (*N* = 36) was put together based on the following criteria: 1) mentioned in at least two separate official reports, 2) suggested by experts and/or stakeholders, 3) having a clear stake in zoonosis risk communication, and/or 4) being a potential end-user of the to-be-developed platform.

*Stakeholder analysis*: We then sent a survey to 20 stakeholders to rate our list of stakeholders using the stakeholder salience approach [15]. According to Mitchell et al. [15], stakeholder salience is made up of three attributes: power, legitimacy, and urgency. We tried to keep our definitions of the three attributes as similar as possible to the definitions van Limburg et al. [12] used in order to keep terminologies consistent. Power was defined as “the level of influence a stakeholder has in the risk communication of zoonoses”; legitimacy was conceptualized as “the level in which a stakeholder needs to be legally, morally, or contractually involved in risk communication of zoonoses”; and finally, urgency was described as “the priority of the stakeholder in risk communication of zoonoses”. Stakeholders were asked to tick a box below each attribute they thought belonged to the stakeholders on the list. It was possible to tick all three boxes, meaning the stakeholder possessed all three attributes. Stakeholders could also tick the boxes “None” meaning none of the attributes were relevant, or “I don’t know”. By adding the latter option, we ensured stakeholders would not leave all the rows blank (indicating they thought the stakeholder did not have power, legitimacy, or urgency) if they did not know the stakeholder well enough to make an informed decision. Only if the majority of stakeholders (>50 %) ticked a box, this was interpreted as a consensus. More information on the survey can be found under ‘value specification’.

### Value specification

Within the value specification phase, key stakeholders are asked what their values (economic, social, and/or behavioral) are and are asked to rate them based on their importance in solving the wicked problem [10]. A value can be defined as ‘an ideal or interest a (future) end user or stakeholder aspires to or has’ (p.5) [27]. With the identification and prioritization of key values, the characteristics of ‘no problem definition’ and ‘no stopping rule’ associated with wicked problems are being tackled. We used a mixed qualitative and quantitative approach in order to identify, rate, and rank relevant stakeholder values. The qualitative interviews we conducted with stakeholders provided input for the survey we set out among these same stakeholders.

*Qualitative interviews:* To identify key values, semi-structured face-to-face interviews were conducted with stakeholders and end-users (*N* = 20) from the three main sectors involved in risk communication of zoonoses in the Netherlands: the veterinary (*N* = 8), human (*N* = 1), and public health (*N* = 11) sectors respectively. One stakeholder was interviewed twice as 1 h had been an insufficient amount of time to capture all the important insights this stakeholder had. Stakeholders ranged from policymakers at governmental level to local general practitioners and veterinarians. They were chosen from our shortened list of stakeholders (*N* = 36) and were selected based on the following criteria: 1) either a hands-on, executive position or a high placed managing director (or similar) position, 2) involved in risk communication and/or decision making regarding zoonoses, 3) preferably a varied background in the context of zoonoses, and 4) willingness and availability to participate. Interviews took mostly place in person (*N* = 19) and some by phone (*N* = 2). Interviews lasted roughly between 30 and 60 min. Questions asked included but were not limited to the current risk communication structures and strategies in the Netherlands and the problems associated with them; stakeholder background, responsibilities and stakeholder salience; tasks of the organization the stakeholder is affiliated with; stakeholder opinions and wishes regarding technology and our to-be-developed platform; and finally, recommendations of other key stakeholders to include (snowball sampling). Questions differed per stakeholder as we wanted to take advantage of each stakeholder’s unique background, viewpoint and experience. Before each interview commenced, written and verbal information was given and written consent was obtained. Ethical approval for this study was granted by the University of Twente Ethics Committee. Interviews were audio recorded and transcribed verbatim. Stakeholders were sent a copy of their interview transcript for validation purposes on request. Inductive thematic analysis was used to code the transcript [28]. Transcripts were entered into NVivo version 10 and coded on the level of meaning units, meaning chunks of data were grouped together based on their joint meaning, irrespective of length. One researcher coded the interviews and created a coding manual. After familiarization with the data by the research team, an expert discussion and evaluation took place to discuss and validate the identified themes and coding manual until consensus was reached.

*Survey:* After extracting key values from the transcripts, a survey was sent to all interviewed stakeholders (*N* = 20) to generate a quantitative inventory of the identified values. Due to the advancement of technology and an increased use of the internet, conducting online survey research has become an attractive way to collect data. Surveys provide access to unique populations and can save the researcher both time and money [29]. Survey research can be of benefit to health professionals and researchers working in medical research areas, especially when the needs and values of individuals are of interest [30]. For an example of a survey in the infection prevention and control context, see [31], where they investigated local implementations of antibiotic stewardship programs in Dutch hospitals. In our study, an online survey is not used as a stand-alone method, but rather as a useful tool to quantify and deepen the data obtained from the qualitative interviews. As such, the research is not a clinical investigation but rather a value-driven one. The online survey is used as a cheap, time-saving and easy tool to quantitatively investigate the level of agreement on the identified key values among our total group of interviewed key stakeholders. The survey was created in Limesurvey and consisted of: a demographic questionnaire consisting of 5 questions, 40 identified values to be rated on a 7-point Likert scale ranging from 1 (very unimportant) to 7 (very important), and 36 stakeholders to be ranked according to the stakeholder salience approach [15]. Stakeholders who did not respond to our email were sent a follow-up email after 1 week reminding them about the survey and kindly requesting them to fill in the survey. If stakeholders failed to respond up until one week after our reminder, we contacted them by telephone.

## Results

### Contextual inquiry

**Fig. 2:**
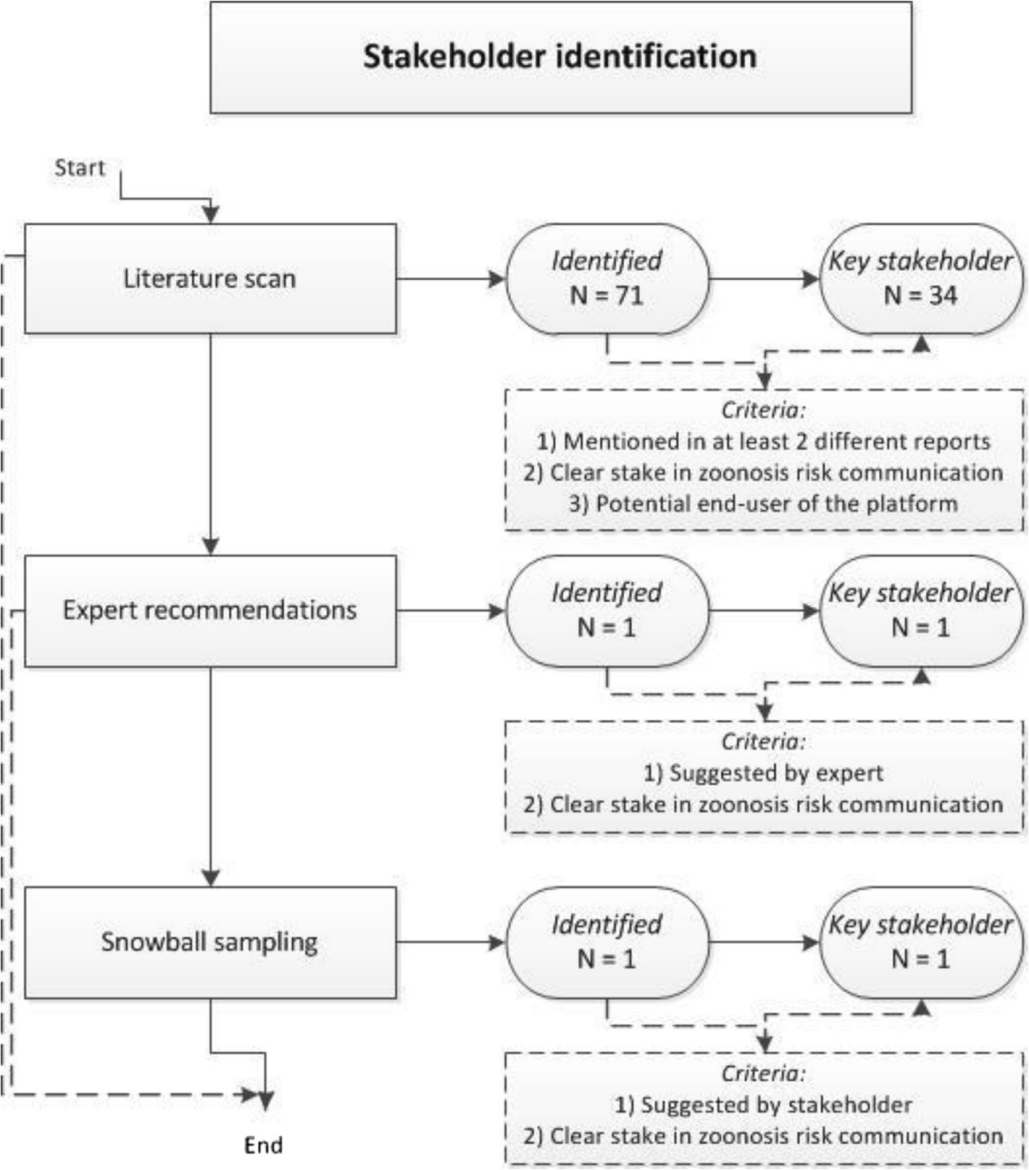
Flowchart illustrating the stakeholder identification process

*Stakeholder identification:* A total of 73 stakeholders were identified across the three sectors of interest (veterinary, human, and public health) and a group of stakeholders who did not neatly fit into one of these sectors (others). For a breakdown of how many stakeholders were identified during the three steps of the stakeholder identification and which selection criteria were used, see the flowchart below (Fig. 2). As can be seen from Fig. 2, the literature scan identified almost all of the key stakeholders. However, expert recommendations and snowball sampling did result in two additional key stakeholders.

*Stakeholder analysis:* We asked stakeholders to rate a shortened version of the complete stakeholder list (consisting of 36 instead of 73 stakeholders) using the stakeholder salience approach. Table 2 provides the stakeholder ratings of our stakeholder list. The overview of stakeholders presented here is essential to find out unofficial opinions of stakeholders about other stakeholders that cannot be found in official reports. In addition, decisions about who to classify as key stakeholders can be made. From Table 2 it can be seen that the majority of all stakeholders were seen as legitimate stakeholders. Even those who did not meet the 50 % mark in order to be classified as legitimate were still seen as legitimate by a subsample of our stakeholders. These high legitimacy scores illustrate a characteristic of wicked problems, namely the relevance of many stakeholders.

**Table 2:** Classification of stakeholders using Mitchell’s stakeholder salience approach

Stakeholders	Power	Legitimacy	Urgency	None	Don’t know
Veterinarian	**54 %**	**85 %**	38 %		
General practitioner	**53 %**	**73 %**	40 %		
Company doctor	20 %	**67 %**	33 %		7 %
Microbiologist	**60 %**	40 %	33 %	7 %	
Infection expert	47 %	47 %	27 %	7 %	7 %
Dutch General Practitioners Society (NHG)	47 %	**60 %**	33 %	13 %	13 %
Royal Dutch Medical Association (KNMG)	40 %	**60 %**	33 %	20 %	7 %
Dutch Society for Medical Microbiologists (NVMM)	**53 %**	40 %	7 %	7 %	20 %
Municipal Public Health Service (GGD)	**85 %**	**62 %**	39 %		
National Institute for Public Health and the Environment (RIVM)	**85 %**	**54 %**	**54 %**		
Dutch Food and Consumer Product Safety Authority (NVWA)	**87 %**	**67 %**	47 %		
Ministry of Health, Welfare and Sport (VWS)	**87 %**	**80 %**	47 %		
Ministry of Economic Affairs (EZ)	**71 %**	**71 %**	43 %	7 %	
Health Care Inspectorate (IGZ)	**57 %**	**50 %**	36 %	7 %	14 %
Netherlands Center for Occupational Diseases (NCvB)	13 %	**67 %**	27 %	7 %	20 %
Dutch Forestry Commission	7 %	47 %	13 %	33 %	20 %
Wageningen University	13 %	47 %	20 %	20 %	20 %
Faculty of Veterinary Medicine, University of Utrecht	29 %	**57 %**	21 %	21 %	7 %
Royal Tropical Institute (KIT)	13 %	40 %	20 %	27 %	13 %
National Information Center Pets (LICG)	7 %	**50 %**	21 %	21 %	14 %
Netherlands Centre for One Health (NCOH)	20 %	**60 %**	20 %	27 %	7 %
GD Animal Health (GD)	**60 %**	**67 %**	33 %	13 %	
Royal Dutch Society for Veterinary Medicine (KNMvD)	33 %	**73 %**	33 %	13 %	7 %
Central Veterinary Institute	39 %	**69 %**	39 %	8 %	8 %
Dutch Wildlife Health Centre	33 %	**53 %**	27 %	20 %	7 %
Dutch Society for Wildlife Health	7 %	40 %	13 %	33 %	20 %
Dutch Federation of Agriculture and Horticulture (LTO)	43 %	**50 %**	43 %	14 %	7 %
Refugees Center		27 %	20 %	47 %	20 %
Farmers	7 %	33 %	13 %	33 %	13 %
Stigas (Health & Safety Service; Agriculture and Horticulture)	13 %	**53 %**	27 %	7 %	40 %
General Public	13 %	33 %	**53 %**	27 %	7 %
World Health Organization	**53 %**	**53 %**	20 %	13 %	20 %
World Organization for Animal Health	**53 %**	47 %	20 %	20 %	20 %
European Centre for Disease Prevention and Control	**60 %**	47 %	27 %	13 %	13 %
Regional knowledge network	43 %	**57 %**	**50 %**	14 %	7 %
Q-Support	7 %	43 %	7 %	14 %	36 %

Power = the level of influence a stakeholder has in the risk communication of zoonoses

Legitimacy = the level in which a stakeholder needs to be legally, morally, or contractually involved in risk communication of zoonoses

Urgency = the priority of the stakeholder in risk communication of zoonoses

Text in bold: >50 % of stakeholders selected this attribute as belonging to the stakeholder on the left

Out of the complete list of stakeholders (*N* = 36), only one stakeholder was considered to possess all three attributes (power, legitimacy, urgency) and was thus seen as a definite stakeholder (National Institute for Public Health and the Environment). 10 stakeholders possessed two attributes, whereas 17 possessed only one. Finally, 8 stakeholders did not possess any attributes. For our zoonoses example case, we found that veterinarians and general practitioners were not perceived as possessing the attribute of urgency, but if they worked together as part of a regional knowledge network, their sense of urgency increased whereas their power dropped (see Table 2). Similarly, farmers had very little power (7 %), but the organization representing them (Dutch Federation of Agriculture and Horticulture) was considered to possess a lot more (43 %).

### Value specification

*Qualitative interviews:* Thematic analysis identified five main stakeholder values and needs that reoccurred across the interviews. According to Braun and Clarke [28], whose approach we followed for our thematic analysis, a theme captures something important in relation to the research question. In our case, only five themes met this criteria. Although three more categories were identified, these were not considered to be themes as they were not perceived to be relevant to the to-be-developed intervention and/or zoonosis risk communication in particular. As a result, these categories were not included in the analysis or reported. The following five themes reoccurred across the interviews and were considered to be relevant after expert evaluation:

- *Collaboration and communication between professionals on a regional level need to be strengthened.* The majority of stakeholders mentioned that there is little to no communication between general practitioners and veterinarians, which has to be improved to ensure the exchange of zoonotic signals and knowledge between the two professionals. For the same reason, the communication of the municipal public health services with other independent services in the local community (e.g. hospitals, nursing homes, veterinarians) has to be improved.

“I think that partnerships can be very meaningful if they start locally. That is the best way, to let it go bottom-up. The other way around is more difficult.” (Veterinary sector)

“We as a municipal public health service had no contact with them [local veterinarians]. As a result of the Q-fever, we thought ‘hey, we should have a lot more contact with them in order to notice things a lot faster.’” (Public health)

(b) *The veterinary and human health sector have different, even conflicting viewpoints regarding problem definitions and solutions of zoonoses prevention and control.* For example, the veterinary sector considers the One Health approach to be of great importance in order to prevent and control zoonoses, whereas the human health sector is not convinced of its importance. Additionally, both sectors define One Health differently and debate about its meaning. Furthermore, the sectors disagree with regards to the amount they want to work together. Veterinarians want more communication and collaboration with general practitioners, whilst general practitioners do not see the advantages of this.

“I talk to many people from the human side of professional organizations … and if I look at what often the working definition of One Health is for a lot of individuals from the human health sector, then actually that is what you should contain in the concept preventative healthcare for humans.” (Veterinary sector)

“Interviewer: Is there something that you notice in comparison to before, before you had contact with the veterinary sector, that it’s advantageous to have those contacts [with the veterinary sector]? Participant: Actually, actually not. Because in my opinion there’s never a massive outbreak of zoonoses in the population. At least not in this area.” (Human health)

(c) *The general public needs to be better informed about zoonoses.* The general public lacks knowledge about what zoonoses are, how they can get infected, and what they can do to prevent infections. As a result, they cannot make accurate risk assessments.

“Look if they [general public] know that eating chicken can make you ill, then you can handle that chicken and you won’t get ill. But these kind of risks from the environment, then they’re like well how does that work, and how does that happen, and if I walk outside does that mean I can get it too? Not only is there a lack of knowledge, but also the assessment of how do you handle these kind of things.” (Public health/Veterinary sector)

(d) * Information needs to be tailored*. Stakeholders made it clear that professionals and general public need to be addressed separately with information tailored to their profiles. Information needs to be simplified for the general public, whereas simplified texts should be avoided to inform professionals as this may cause frustration and confusion.

“It is of course always difficult, and it’s always taught that you should focus on your target population. If you make something for both [general public and professionals] at once, then you get a one size fits all/one size fits nobody so to speak. Then it’s suitable for neither.” (Public health, Veterinary sector)

(e) *The attitude of both general public and professionals needs to be changed*. Currently, the general public and the human health sector underestimate the urgency and consequences of zoonoses which leads to several negative consequences. According to professionals, the general public sees advice about preventing zoonoses as overbearing and as a result do not act on this information. Secondly, general practitioners are not interested in communicating and collaborating with local veterinarians, which results in the conflicting views described previously. An attitude change for both general public and human health professionals is warranted to ensure better preventative actions by these groups.

“And you don’t allow that dog to lick the kids’ bowl or take the pacifier in its mouth etcetera etcetera. You need to be aware as parents that that is not healthy, and that you shouldn’t evoke it, and please stop shouting all the time because they all do ‘but we are already very hygienic, so you need to build resistance’.” (Veterinary sector)

“And no matter what we do to invite them [general practitioners] to meetings for example, they just don’t show up. They don’t find that [zoonoses] interesting, don’t want to know anything about it, because they don’t see them so they’re not important.” (Veterinary sector).

*Survey:* The five overarching themes discussed above were made up of individual codes (chunks of information grouped together based on their joint meaning). Two researchers scanned the individual codes for inclusion into the survey as an independent value. The criteria for selection were codes referring to 1) high complex situations or dilemmas (a wicked context), 2) One Health contexts, 3) problems in current practice, 4) opinions on which stakeholders could disagree, and 5) the to-be-developed platform. In addition, codes that were mentioned in more than one interview were preferred. Critical evaluation of codes led to the inclusion of 40 items, which were evaluated across the research team and deemed to be a representative selection of the qualitative data. We sent out a survey to let stakeholders weigh and prioritize the identified values. Out of all stakeholders (*N* = 20) who were sent the survey, we had a response rate of 75 %. Out of the five stakeholders who did not fill in our survey, three said they were too busy, and the research team was unable to get a hold of the remaining two participants. The average age of stakeholders was 51 years old (SD = 8.91). They had worked in the field of zoonoses for an average of 12 years (SD = 8.06). The five highest-scoring values regarding the development of our eHealth intervention were that the innovation needs to (A) provide information coming from a trustworthy source, (B) be connected with existing zoonoses-initiatives, (C) contain links to other websites about zoonoses, (D) provide relevant information, and (E) provide up-to-date information. In our case, the majority of values were perceived to be important by the key stakeholders and were rated highly. In the context of wicked problems, those items are not the ones that need to be looked at by the researcher as there is already consensus among stakeholders. It is more important to look at items on which stakeholders have opposing views and subsequently rate very differently, as these items need to be addressed properly in order to avoid unsupportive stakeholders later on. On 67.5 % (*N* = 27) of the items, stakeholder responses fell between ‘not important, not unimportant’ and ‘very important’. On the other items the distribution of responses varied significantly, with some stakeholders rating items as ‘very unimportant’ whilst others rating this same item as ‘very important’. For example, the item ‘the innovation needs to have a closed off area for professionals’ responses ranged from ‘unimportant’ to ‘very important’. Items like this one are important to recognize as consensus needs to be reached on these issues before the innovation can be developed further. Seeing as wicked problems consist of many different stakeholders with different or even opposing views, a depiction of the overall preferences only would not suffice as we might unintentionally favor the opinions of some groups over others. Due to the inclusion of stakeholders who did not belong to either group (e.g. a stakeholder working at a regional knowledge network for zoonoses), or were currently employed in multiple sectors (both veterinary and public health) or had a history of switching domains (now working in public health, but previously employed in the veterinary sector), and the underrepresentation of stakeholders in the human health sector, we divided all stakeholders into two groups: a group of stakeholders with a background in the veterinary sector (*N* = 6), and a group of stakeholders with a background in the public and/or human health sector (*N* = 9). Findings suggest that although differences exist between both sectors, there are similarities as well. As can be seen from Table 3, both sectors have a need for information coming from a trustworthy source and want the innovation to be connected to other zoonosis-initiatives and websites. Differences between the sectors were evident too, for example a key value from the veterinary field (provide information proactively in times of outbreak) did not make it to the key values of the human/public health sector.

**Table 3:** Overview of the 5 highest scoring values grouped by sector (veterinary and human/public health)

Value	
The intervention needs to…	
Veterinary sector	
Provide information proactively in times of outbreak	
Provide information coming from a trustworthy source	
Tune the information on national level and local level with each other	
Contain links to other websites about zoonoses	
Have connections with existing zoonosis-initiatives	
Human/Public health sector	
Have connections with existing zoonoses-initiatives	
Provide information coming from a trustworthy source	
Provide relevant information	
Contain links to other websites about zoonoses	
Provide up-to-date information	

## Discussion

In this article, we devised a step-by-step guideline for co-creating with stakeholders when tackling wicked problems based on prior research in infection prevention and control and an existing stakeholder-centered approach [12], and we demonstrated the use of this guideline by an illustrative zoonoses example case.

Following this guideline provides the researcher with a systematic, transparent approach of how to work with stakeholders, and this approach was proven useful to gain insights from multiple perspectives into the context of the wicked problem. In doing so, the three characteristics associated of working with wicked problems (no agreement about problem definition and solution, involvement of many stakeholders with strong-held conflicting views, and no clear end-point of the problem-solving process) are dealt with. This study is the first to provide a practice-based guideline for tackling wicked problems in the field of human-animal interactions, and other wicked problems more generally. Furthermore, it adds to existing literature by testing the effectivity of an established holistic framework for involving and collaborating with stakeholders in eHealth research [9, 10, 12, 13, 27] in the context of a wicked problem.

Some might argue that it takes a lot of time and effort to follow this guideline. However, the time needed to work with this guideline depends on how many stakeholders the research team wants to involve, bearing in mind that certainly not all stakeholders need to be included [8]. Others might worry that stakeholders do not have enough time to collaborate extensively with the research team, and although planning and preparing can be time-consuming for the researcher, participation only minimally burdens the involved stakeholders. For the stakeholder, only the time it takes to give an interview or fill in a questionnaire is needed. A way to limit the amount of time invested by the researcher is to find practical ways to work with experts and stakeholders, such as using an online panel to collect data. For example, a Delphi panel [32] is a less time-consuming choice. However, we expect potential benefits such as increased stakeholder commitment due to personal encounters to be smaller when using online communication only. Testing this hypothesis provides an interesting avenue for future research. Another example of a less time-consuming choice is organizing focus groups instead of one-on-one visits, but this way of working is not always feasible due to busy schedules of invited participants. Although this study focused on involving stakeholders for eHealth development, stakeholders are being consulted in other settings as well. In the field of zoonoses, examples are decision making studies to identify criteria used for prioritization of zoonoses in Canada [33] and Japan [34]. In yet another study, a stakeholder approach was used to identify current gaps of knowledge and research priorities in the area of infection control and occupational health [35]. We see no reason as to why this framework would not be suitable to use in settings outside of eHealth development. In particular, we see possibilities for this approach being used by research teams of hospitals or governmental organizations as a way to explore the context before ‘diving in’. We call upon future research to test the suitability of this guideline outside of eHealth research.

This study has some limitations that should be taken into account. Firstly, results of this study are unable to prove the effectiveness of this approach in tackling wicked problems (yet) as we have not implemented our platform yet. Identifying stakeholder values and subsequently implementation requirements is only the first step in the process.

Secondly, a possible sample bias may have occurred as human health professionals were underrepresented in this sample. We believe the human health professionals were underrepresented as only one stakeholder had a background solely in human health – the other stakeholders mostly had a background mixed in human health and public health. We experienced difficulty with recruiting human health professionals, as many did not respond to our invitation to take part or politely declined. We believe this shows how difficult it is to get stakeholders together when dealing with wicked problems. In addition, the division of stakeholders in groups was complex due to the often varied backgrounds of the stakeholders, which meant they did not neatly fit in one of the sectors. This may have caused a distortion of the results. However, a (potential) sample bias would not reduce the importance of the mentioned key values, as they are brought forward by relevant stakeholders. Other than this possible sample bias, we have no reason to assume any other form of bias occurred from either survey design or responses.

Thirdly, the guideline leaves the research team with a considerable amount of freedom to make choices. Subjective decisions include but are not limited to which stakeholder analysis method to use or what should be the content of the interviews.

Finally, though the setting in which this guideline has been created could be seen as a limitation since it is based on data from one country only, the authors see no reason to assume that the proposed guideline cannot be used in other countries as well. Yet, further research from researchers based in other countries following this guideline is needed to test the generalizability of this approach outside of the Netherlands. In future research, we plan to (A) further design, evaluate, and implement the eHealth innovation (eZoon platform) following the CeHRes road map, and (B) apply this guideline in other research cases. In doing this, we aim at contributing to the evidence-based knowledge and practice of using co-creation with stakeholders to tackle wicked problems. As a next step for our current research, we will continue to iteratively involve end-users and field experts throughout the whole design process, which will guide the further development of the eZoon platform. Further research is needed to validate this guideline and to investigate which approaches work best for co-creating with stakeholders when tackling wicked problems.

## Conclusions

Involving and collaborating with stakeholders in eHealth development and other settings is still a relatively new approach that needs further exploration, especially in the context of wicked problems. The guideline we applied provides a structured, transparent way of involving stakeholders in research projects. These findings contribute to the further development of guidelines on how to work with stakeholders when tackling wicked problems.

### Ethics approval and consent to participate

Before each interview commenced, written and verbal information was given and written consent was obtained from each participant. Ethical approval for this study was granted by the University of Twente Ethics Committee, project number 14234.

### Consent for publication

Not applicable.

### Availability of data and materials

The datasets supporting the conclusions of this article are available from the authors on request to any scientist wishing to use them for non-commercial purposes.

## Abbreviations

CeHRes: Center for eHealth Research
